# A Case of Hand-Foot-and-Mouth Disease in an Adult Male

**DOI:** 10.7759/cureus.42670

**Published:** 2023-07-29

**Authors:** Ashwin Jagadish, Abhijith Paladugula, Shahnawaz Notta, Nasir Notta, Rupal Shah

**Affiliations:** 1 Internal Medicine, East Tennessee State University James H. Quillen College of Medicine, Johnson City, USA

**Keywords:** comprehensive physical exam, viral infection, adult internal medicine, hand-foot-mouth disease in adults, hand-foot-mouth disease

## Abstract

Hand-foot-and-mouth disease (HFMD) is commonly seen in infants and children; less frequently, it may be seen in adults as well. The disease is usually associated with viral infections, including many variants of enteroviruses and coxsackieviruses. We discuss the case of a 39-year-old male who presented with constitutional symptoms, fever, and lesions on his hands, feet, and mouth. His children, who had been recently diagnosed with HFMD, were likely the source of his infection. A comprehensive history and physical examination enabled us to identify the lesions, some of which were faint and difficult to visualize. Viral panel testing indicated positive results for human rhinovirus/enterovirus. Treatment and testing associated with the patient’s condition were supportive, largely based on the history and physical findings which helped us narrow down our differential diagnoses. Complete resolution of the symptoms within one to two weeks is generally expected in these patients.

## Introduction

Hand-foot-and-mouth disease (HFMD) is a viral disease often caused by enterovirus or coxsackievirus [1]. In the United States, coxsackievirus A6 is the leading cause of HFMD [2]. The infection is most commonly seen in children under the age of 10 years [3]; however, one large surveillance study has reported that more than 90% of cases occur in individuals aged less than five years [2]. Typical symptoms of HFMD include malaise, fever, decreased appetite, pain in the oral cavity, and lesions involving the hands, feet, oral cavity, upper extremities, lower extremities, and buttocks [2].

HFMD is transmitted from human to human via direct inoculation from bodily secretions such as saliva, stools, or nasal secretions [1]. Once contact happens, the virus is implanted in the mucosa of the mouth or ileum, from where it then spreads to the bloodstream [1]. Lymphatic involvement typically occurs after 24 hours of presence in the blood [1]. Elimination from the respiratory system may take up to three weeks, while elimination from the gastrointestinal system may take up to eight weeks [1].

## Case presentation

**Figure 1:**
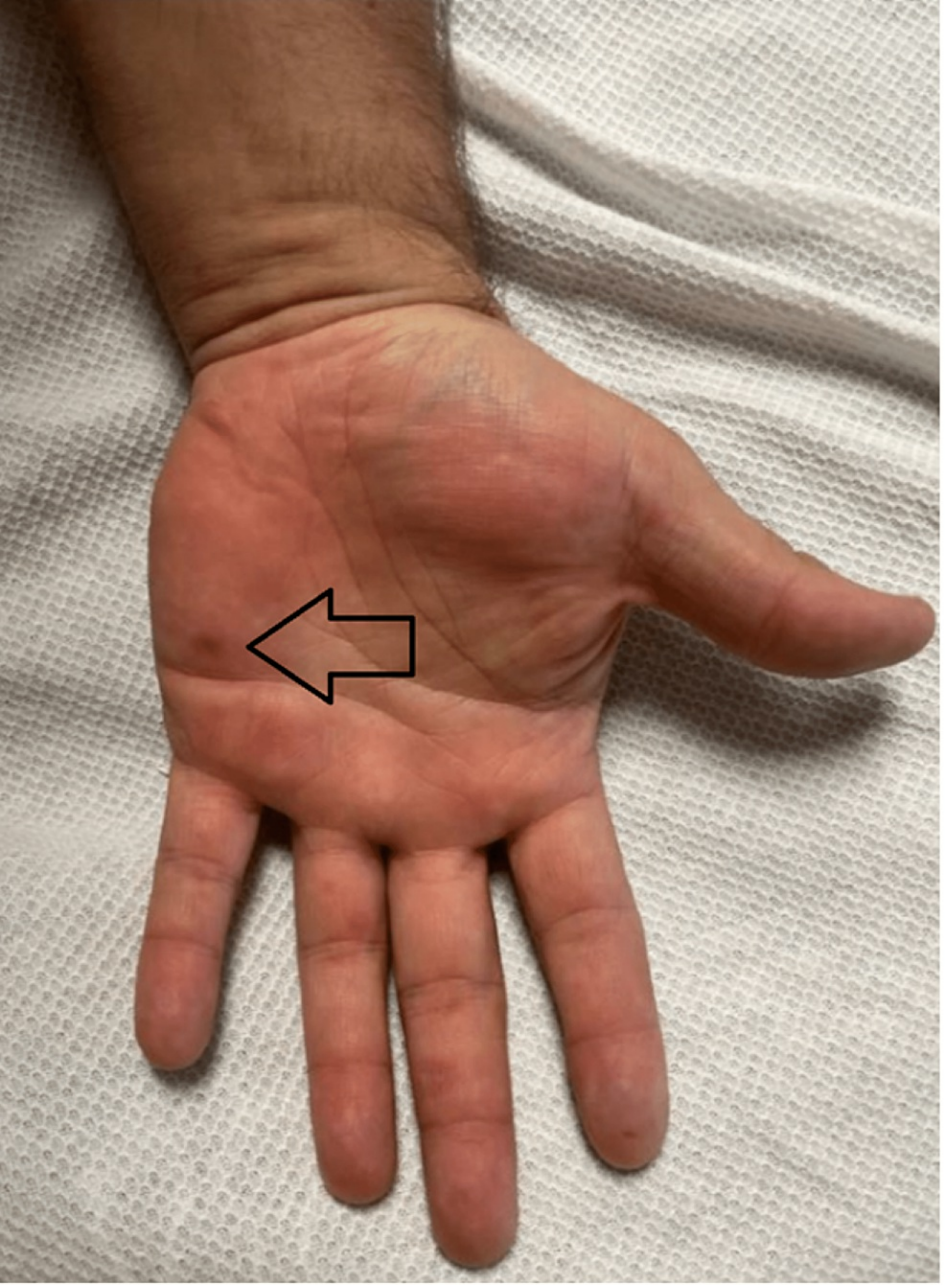
Image of the lesion on the patient's hand The arrow indicates a lesion on the patient's hand that was attributed to hand-foot-and-mouth disease

**Figure 2:**
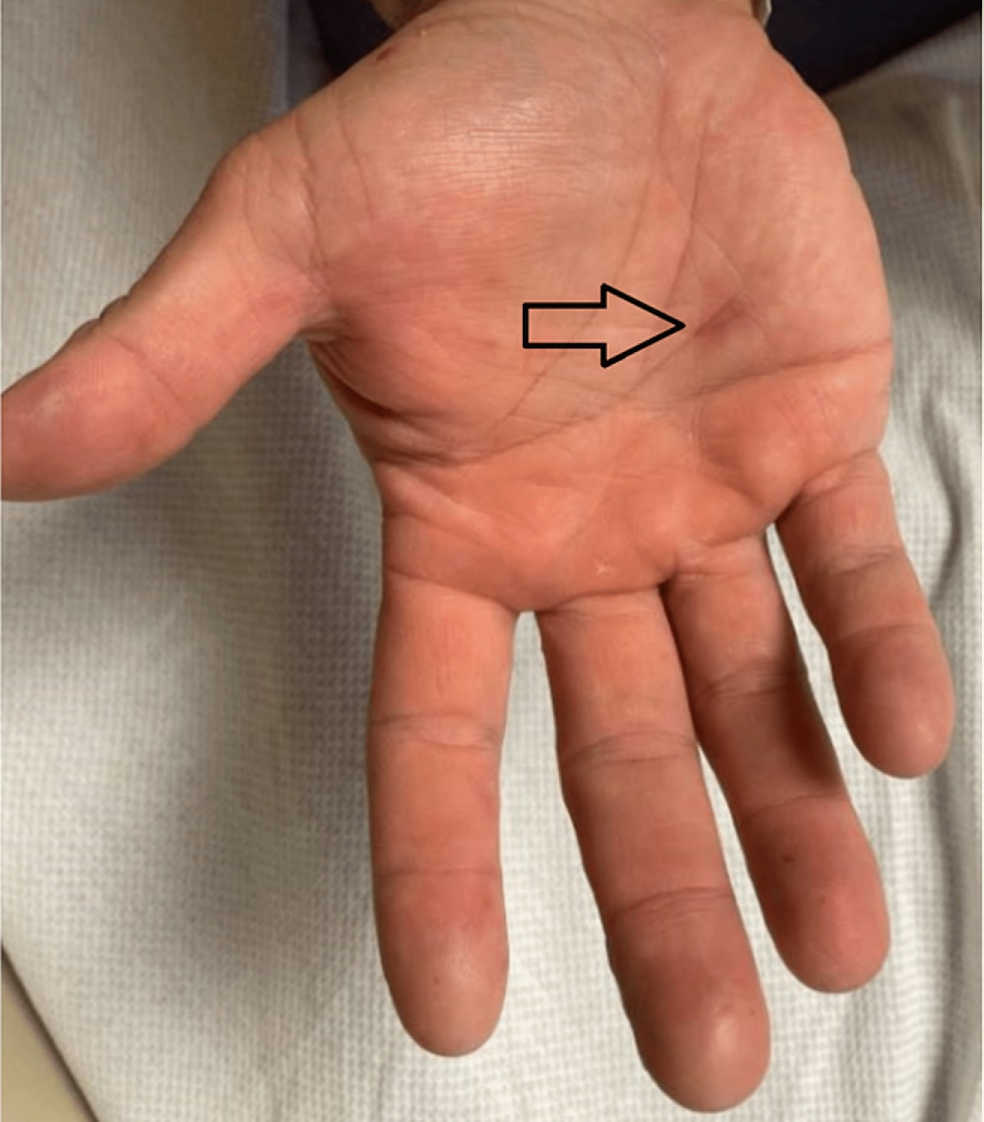
Image of the lesion on the patient's other hand The arrow indicates a lesion on the patient's other hand that was attributed to hand-foot-and-mouth disease

**Figure 3:**
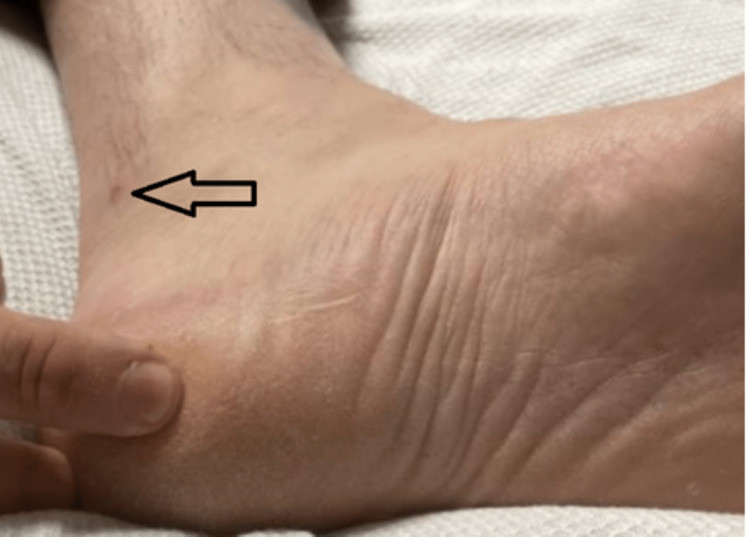
Image of lesion on the patient's foot The arrow indicates a lesion that was attributed to hand-foot-and-mouth disease

A 39-year-old male with a past medical history of hypertension, obstructive sleep apnea, and hyperlipidemia presented to the emergency department due to shortness of breath and self-reported fever. The patient reported having a maximum axillary temperature of 100.5 °F when tested at home. In addition, he endorsed pain with swallowing, pain in his hands and feet, difficulty sleeping, right-sided retro-orbital and temporal headaches, nausea, dry retching, exertional dyspnea, and chest pain. The patient denied having exertional dyspnea and chest pain prior to this episode. The patient’s children had recently been diagnosed with HFMD. A chest X-ray demonstrated no acute cardiopulmonary changes, and a respiratory viral panel was positive for human rhinovirus/enterovirus. Physical examination revealed healing lesions on the patient’s hands, feet, and mouth. The lesions of the hands can be seen in Figures 1-2, and the lesions of the feet can be seen in Figures 3. The patient requested that images of his oral cavity not be taken. The patient’s treatment was supportive, consisting of oral hydration, as well as acetaminophen and ibuprofen as needed.

## Discussion

Our patient presented with general symptoms including fever, chills, headaches, exertional dyspnea, and chest pain. Further questioning revealed difficulty in swallowing and pain in his hands and feet. Physical examination revealed healing lesions in his oral cavity and on his hands and feet. At the time of presentation, there were a few visible lesions; some were faint and a thorough examination was needed to identify them. Additionally, he tested positive for rhinovirus/enterovirus. These findings contributed to the diagnosis of HFMD.

The classical presentation of HFMD involves fever and vesicular lesions that are restricted to the hands, feet, and oral cavity [4]. However, the characteristics of HFMD in adults can be atypical or severe [4]. The fever may be higher; also, lesions may have differing appearances and be located in the arms, legs, and trunk as well [4]. Atypical cases are often diagnosed based on history and physical examination [4]. The diagnosis may be supported by immunoenzyme assays or gene amplification testing [4].

Treatment of HFMD is generally supportive; hydration is important, and pain management can be achieved with acetaminophen or ibuprofen [2]. The prognosis is usually good with complete resolution within one to two weeks, and recurrence is rare [2]. Sometimes, serious complications may arise. These include stomatitis with painful ulcers that may affect oral intake, aseptic meningitis associated with enterovirus 71, or pulmonary edema, myocarditis, spontaneous abortion, interstitial pneumonia, and pancreatitis associated with coxsackievirus [2].

A rise in HFMD cases may occur in the future due to an increase in international travel, the evolution of viruses, and global temperature changes [5]. However, there are currently no medications or vaccines available for this condition [6]. Thus, preventative measures and education regarding hygiene are important to limit the spread of HFMD [6].

## Conclusions

While HFMD is a disease that primarily impacts children, it may be seen in adults as well. Patients who present with nonspecific symptoms of fever, chills, and headache should receive a thorough physical examination to evaluate for lesions. The presence of lesions on the hand, feet, and oral cavity, even if few or faint, may help diagnose HFMD if accompanied by a positive test for human rhinovirus/enterovirus. Treatment of HFMD is generally supportive, and the prognosis tends to be good with complete resolution achieved within weeks. Given the lack of definitive treatment options currently, prevention and education are keys to limiting the spread of HFMD.
